# Supplementary material for: Entomopathogenic Fungi Effectively Control Phorodon cannabis Aphid Population in Cannabis sativa Plants
Source: Plants (Basel). 2025 Mar 16;14(6):931. doi: 10.3390/plants14060931 (PMC11946862; doi:10.3390/plants14060931)
Supplement: Supplementary file 1 [file plants-14-00931-s001.zip › plants-3503334-supplementary.pdf]

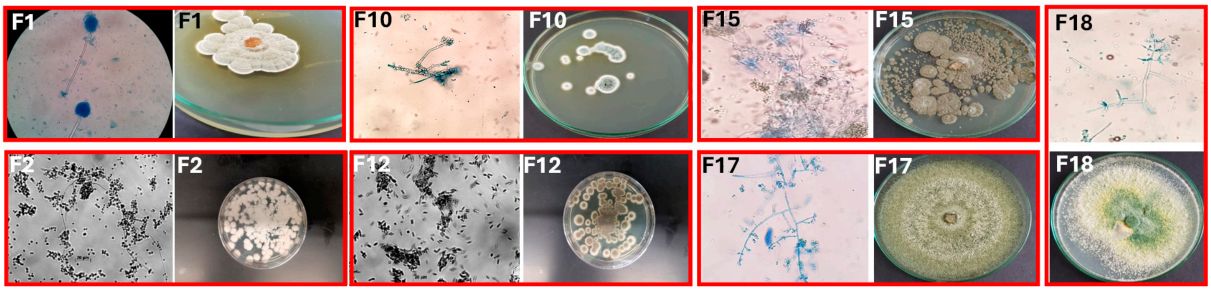

Supplementary Figure S1. Morphology of isolated colonies. Isolate F2 exhibited a white to pale cream color with a cottony or powdery texture. Under the microscope, after staining with lactophenol blue, F2 showed small, single-celled, oval or globose conidia produced on short, zig-zag conidiophores. The conidia were often arranged in dense clusters, giving the colony a globular appearance. Isolate F12, on the other hand, appeared green to olive in color with a granular texture. After two weeks of incubation on PDA, the colonies developed a powdery appearance due to conidia formation. Microscopic ex-amination revealed that the conidia of F12 were cylindrical to elliptical and were borne on distinct, branched conidiophores, typically arranged in dense, brush-like clusters.

Supplementary Table S1. Total terpenes concentration (g) of Congo durban (THC dominant) cannabis plants using USP <621> chromatography and HPLC-DAD quantification. Bb, *B. bassiana* 1 x10^{7} conidia ml^{-1}.

Terpenes	Ct	I-W	I-I	I-B	N-B
**a-Pinene**	2.81	2.33	2.86	3.14	2.79
**b-Myrcene**	0.50	0.47	0.60	0.58	0.49
**b-Pinene**	1.80	1.63	1.54	2.48	1.86
**b-Caryophyllene**	0.09	0.10	0.01	0.11	0.00
**Farnesene**	0.48	0.45	0.48	0.45	0.46
**δ-Limonene**	0.00	0.00	0.09	0.00	0.09
**a-Cedrene**	0.03	0.00	0.00	0.03	0.00
**a-Bisabolol**	0.10	0.12	0.10	0.13	0.12
**Terpineol**	0.11	0.08	0.09	0.13	0.17
**Linalool**	0.00	0.00	0.00	0.00	0.05
**trans-Nerolidol**	0.86	0.00	0.68	1.25	0.88
**Isoborneol**	0.06	0.90	0.05	1.33	0.05
**Valencene**	0.04	0.00	0.35	0.00	0.27
**cis-Nerolidol**	0.00	3.67	0.00	5.23	0.00
**Ocimene**	0.00	0.02	0.00	0.02	0.06
**Geranyl Acetate**	0.35	0.41	0.30	0.48	0.31
**(+)-Pulegone**	0.02	0.05	0.00	0.00	0.04
**Camphene**	0.00	0.00	0.03	0.00	0.00
**Camphor**	0.02	0.00	0.00	0.00	0.00
**Cedrol**	0.02	0.02	0.02	0.02	0.03
**Eucalyptol**	0.64	0.16	0.51	0.16	0.55
**Fenchol**	0.00	0.02	0.00	0.02	0.02
**Fenchone**	1.63	0.00	1.75	0.02	1.82
**ϒ-Terpinene**	0.03	0.02	0.00	0.02	0.02
**Geraniol**	0.02	0.02	0.00	0.00	0.00
**Total**	9.62	10.48	9.45	15.60	10.08

Supplementary Table S2. Total terpenes concentration (g) of Perseid (CBD dominant) cannabis plants using USP <621> chromatography and HPLC-DAD quantification. Bb, *B. bassiana* 1 x10^{7} conidia ml^{-1}.

**Terpenes**	**Ct**	**I-W**	**I-I**	**I-B**	**N-B**
**a-Pinene**	0.53	0.01	0.30	0.80	0.77
**b-Myrcene**	0.34	0.02	0.13	0.36	0.42
**b-Pinene**	0.21	0.02	0.12	0.31	0.30
**Guaiol**	0.15	0.06	0.02	0.14	0.19
**b-Caryophyllene**	0.11	0.02	0.09	0.17	0.18
**Farnesene**	0.11	0.00	0.08	0.16	0.18
**δ-Limonene**	0.11	0.00	0.03	0.12	0.15
**a-Cedrene**	0.11	0.00	0.00	0.04	0.11
**a-Bisabolol**	0.06	0.00	0.02	0.03	0.10
**Terpineol**	0.10	0.00	0.08	0.10	0.10
**Linalool**	0.07	0.00	0.05	0.06	0.07
**trans-Nerolidol**	0.07	0.02	0.01	0.04	0.07
**Isoborneol**	0.04	0.27	0.00	0.00	0.06
**Valencene**	0.02	0.00	0.00	0.01	0.06
**cis-Nerolidol**	0.00	0.00	0.00	0.02	0.05
**Ocimene**	0.00	0.00	0.02	0.00	0.02
**Geranyl Acetate**	0.02	0.00	0.01	0.05	0.01
**(+)-Pulegone**	0.01	0.00	0.00	0.00	0.01
**Camphene**	0.02	0.00	0.02	0.00	0.01
**Caryophyllene Oxide**	0.00	0.00	0.01	0.00	0.00
**Cedrol**	0.00	0.00	0.01	0.01	0.00
**Eucalyptol**	0.02	0.00	0.00	0.01	0.00
**Fenchol**	0.00	0.00	0.10	0.12	0.00
**Geraniol**	0.00	0.00	0.04	0.01	0.00
**Isopulegol**	0.04	0.00	0.00	0.03	0.00
**Menthol**	0.02	0.00	0.00	0.01	0.00
**Nerol**	0.00	0.02	0.01	0.00	0.00
**p-Cymene**	0.00	0.00	0.00	0.01	0.00
**Total**	2.12	0.44	1.19	2.64	2.87

Supplementary Table S3. Total terpenes concentration (g) of GCC (CBD ≈ THC) cannabis plants using USP <621> chromatography and HPLC-DAD quantification. Bb, *B. bassiana* 1 x10^7 conidia ml^{-1}.

**Terpenes**	**Ct**	**I-W**	**I-I**	**I-B**	**N-B**
**a-Pinene**	0.41	0.64	0.12	0.34	0.59
**b-Myrcene**	1.27	2.24	1.01	1.90	2.09
**b-Pinene**	0.28	0.39	0.17	0.38	0.33
**Guaiol**	1.12	0.93	0.67	0.96	0.93
**b-Caryophyllene**	0.29	0.33	0.26	0.24	0.25
**Farnesene**	0.73	0.59	0.47	0.64	0.50
**δ-Limonene**	0.38	0.65	0.32	0.55	0.56
**a-Cedrene**	0.23	0.23	0.00	0.24	0.04
**a-Bisabolol**	1.98	0.44	0.34	0.36	0.48
**Terpineol**	0.19	0.25	0.14	0.33	0.24
**Linalool**	0.22	0.28	0.12	0.25	0.32
**trans-Nerolidol**	0.02	0.04	0.04	0.00	0.01
**Isoborneol**	0.13	0.06	0.12	0.13	0.05
**Valencene**	0.05	0.24	0.18	0.05	0.25
**Ocimene**	0.00	0.03	0.00	0.00	1.46
**Geranyl Acetate**	0.09	0.03	0.00	0.08	0.05
**(+)-Pulegone**	0.04	0.14	0.06	0.00	0.00
**Camphene**	0.03	0.02	0.01	0.05	0.04
**Cedrol**	0.02	0.00	0.00	0.03	0.00
**Eucalyptol**	0.03	0.02	0.07	0.03	0.04
**Fenchol**	0.00	0.00	0.21	0.00	0.28
**Fenchone**	0.00	0.00	0.00	0.00	0.02
**ϒ-Terpinene**	0.16	0.28	0.19	0.20	0.00
**Geraniol**	0.02	0.00	0.10	0.00	0.04
**Isopulegol**	0.00	0.00	0.00	0.00	0.00
**Menthol**	0.00	0.00	0.03	0.00	0.02
**Nerol**	0.00	0.00	0.03	0.00	0.00
**Total**	7.68	7.85	4.67	6.76	8.60
